# Clinical Cancer Research in South America and Potential Health Economic Impacts

**DOI:** 10.3390/healthcare11121753

**Published:** 2023-06-15

**Authors:** William de Oliveira Avellar, Édria Aparecida Ferreira, Ana Carolina Rodrigues Alves Vieira, Andreia Cristina de Melo, Veronica Aran

**Affiliations:** 1 Division of Clinical Research and Technological Development, Brazilian National Cancer Institute (INCA), Rua André Cavalcanti 37, Bairro de Fátima, Rio de Janeiro 20231-050, Brazil; avellarnetto@gmail.com (W.d.O.A.);; 2 Instituto Estadual do Cérebro Paulo Niemeyer (IECPN), Rua do Rezende, 156-Centro, Rio de Janeiro 20231-092, Brazil

**Keywords:** cancer, clinical trials, cancer epidemiology, research funding, innovation, health economics

## Abstract

Background: Increased global cancer incidence rates have led to a growing demand for cancer diagnosis and treatment, as well as basic and clinical research on the subject. The expansion of clinical cancer trials beyond the borders of highly developed countries has aided the arrival of these assessments in South American countries. In this context, this study’s objective is to highlight clinical cancer trial profiles developed and sponsored by pharmaceutical companies and conducted in South American countries from 2010 to 2020. Methods: This study comprises descriptive and retrospective research conducted following a search for clinical trials (phases I, II and III), registered at clinicaltrials.gov, carried out in Latin American countries and sponsored by pharmaceutical companies (“Argentina”, “Brazil”, “Chile”, “Peru”, “Colombia”, “Ecuador”, “Uruguay”, “Venezuela”, “Paraguay”, “Bolivia”), registered between 1 January 2010 and 31 December 2020. A total of 1451 clinical trials were retrieved, of which 200 trials unrelated to cancer were excluded and 646 duplicates were removed, leading to a final total of 605 clinical trials employing qualitative and quantitative analyses. Results: A 122% increase in the number of clinical trial registrations from 2010 to 2020 was noted, with a prevalence of phase III studies (431 trials of a total of 605). Lung (119), breast (100), leukemia (42), prostate (39) and melanoma (32) were the main cancers tested for new drugs. Conclusions: The data reported herein indicate the need for strategic basic and clinical research planning that considers South American epidemic cancer profiles.

## 1. Introduction

Cancer is a global public health problem that significantly affects social and economic aspects of human populations and, consequently, of countries. Studies estimated 19.3 million new cancer cases and 10 million deaths worldwide in 2020, with a substantial associated socioeconomic burden, and 170 million years of healthy life lost annually to the disease, with an associated economic cost of USD1 trillion [1]. The increased incidence of chronic diseases, population aging and rising costs due to health care complexity have aggravated this situation [1]. For example, cancer is responsible for high morbidity and mortality rates in Latin America and the Caribbean, following worldwide cancer distributions to some extent [2,3]. GLOBOCAN 2020 data on South American cancer incidence and mortality rates indicate that the sum of the ten most frequent types of cancer is 714,380 new cases, comprising 65% of the total cases (1092.578). Slightly over 50% of deaths were due to lung, colorectal, breast, stomach, prostate and pancreas cancers. Table 1 summarizes the top 10 most common cancers in 10 South American countries in 2020, in addition to all other cancers combined in these countries.

Given this scenario, we can note a clear need for health investments to accompany the growing demand for cancer treatments and diagnosis, which have, up to now, been carried out and financed by large pharmaceutical companies affiliated with more developed countries. National efforts in partnership with the international productive sector to develop sustainable research infrastructure and the dissemination of cancer prevention and care measures in countries undergoing demographic transitions, especially low- and middle-income countries such as Brazil, are paramount to the control and management of this disease.

Basic and clinical research expand both cancer population control policies and interactions between the industrial sector and health services associated with the development of new therapeutic technologies. The development of a new drug comprises different steps to assess its safety and efficacy through human trials based on clinical research activities. Clinical trials include phases I to IV, which aim to confirm potential benefits and adverse events associated with the investigated drugs. It takes approximately 10 years or more for a new drug to be considered suitable for commercialization [5].

Previously restricted to countries of high economic and social development levels, a global expansion of multicentric clinical trials beyond the borders of North America and Western Europe took place during the 2000s based on the need to expand drug assessments in different ethnic groups distributed worldwide and thus expand product commercialization [6].

Clinical research centers comprise an integral part of the research and innovation process designed to advance and improve disease treatments and prevention, operating on the frontier between the development of new pharmaceutical products and health service activities. Clinical research thus becomes a connection and a convergence point that involves pharmaceutical companies’ interests, on the one hand, and regional South American health service and research center needs, on the other.

Under this configuration, clinical research investment patterns must carefully observe demographic and epidemiological transition processes. Evidence concerning the prevalence of diseases in certain populations is important for health sector decision-making processes. The processes of making relevant decisions and setting appropriate priorities should be guided by public policy makers, who should be informed about the scale of specific population health problems, including groups displaying risks and health status trends over time.

Efforts associated with public health, which have increasingly focused on health promotion and disease prevention, can complement clinical care to provide comprehensive health services that promote disease prevention and cures. However, an increasing number of health systems, not only in Brazil but also in other countries, are facing pressures that result in low performance and growing care inequalities [7].

One alternative that may be used to address pressures and achieve positive changes that prioritize population well-being is the analysis of the public health profiles of specific populations to identify their needs and guide the provision of care and services that effectively serve these populations [8]. For example, one study conducted in Belgium indicated that routinely quantifying illness levels in terms of Disability-Adjusted Life Years (DALYs) provides significant added value to evidence-based public health policies in that country [9].

In this context, analyses of clinical cancer trial profiles sponsored by pharmaceutical companies in South American countries become paramount to obtain answers associated with research financial investment patterns for the treatment of diseases that affect local populations.

## 2. Materials and Methods

### 2.1. Study Type

This is a descriptive and retrospective study aiming to assess the clinical trial profiles of studies that evaluated the efficacy and safety of novel drugs focused on cancer treatment, developed and sponsored by pharmaceutical companies in South American countries. The study evaluated the period from 2010 to 2020.

### 2.2. Eligibility

The following filters were applied for the search performed on clinicaltrials.gov (for each country separately): Condition or Disease (“cancer”); Study type (“interventional”); Age, years (child: birth–17; adult: 18–64; and older adult: 65+); Country (“Argentina”, “Brazil”, “Chile”, “Peru”, “Colombia”, “Ecuador”, “Uruguay”, “Venezuela”, “Paraguay”, “Bolivia”); Phases (“I”, “II” and “III”); Funder type (“industry”); and Study period: from 1 January 2010 to 31 December 2020. The search was concluded on 19 October 2021.

The inclusion criteria were clinical trials evaluating the efficacy and safety of drugs applied to cancer treatments sponsored by pharmaceutical companies. The exclusion criteria included trials where the main objective was the diagnosis or management of cancer-related or treatment-related symptoms.

### 2.3. Detailed Search Method and Analysis

Data were obtained from the clinicaltrials.gov registration database (all publicly accessible), tabulated in an electronic spreadsheet in the Excel program and analyzed using descriptive statistics. The initial search identified 1451 clinical trials (Brazil 473, Argentina 332, Chile 193, Peru 132, Colombia 111, Ecuador 5, Uruguay 2, Venezuela 2, Paraguay 1). A total of 200 non-cancer studies were excluded, and 646 duplicates were removed, with 605 clinical trials remaining for the qualitative and quantitative analyses. Content identification, differentiation and grouping were blindly and independently conducted by three researchers to avoid bias and guarantee a higher-quality assessment [10].

The analyses and inferences presented in the discussion were associated with the South American cancer burdens indicated by GLOBOCAN 2020 [4].

Researchers interested in the complete database extracted from both GLOBOCAN 2020 and clinicaltrials.gov are welcome to contact us. All the information will be provided upon request.

## 3. Results

### 3.1. South American Clinical Cancer Trial Profiles

The profiles of cancer clinical trials registered at clinicaltrials.gov from 2010 to 2020 are shown in Table 2, represented as the number of clinical trials sponsored and developed by pharmaceutical companies in South American countries.

### 3.2. Studies Sponsored by Pharmaceutical Companies in South American Countries (2010–2020)

**Figure 1:**
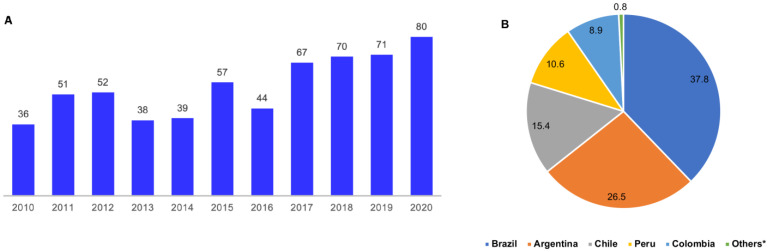
Registrations concerning new international multicenter clinical trials sponsored by pharmaceutical companies in South American countries from 2010 to 2020. (**A**) Number of new cancer clinical trials registrations per year in South America, 2010–2020. (**B**) Percentage of new cancer clinical trial registrations per country in South America, 2010–2020: * Ecuador (5), Uruguay (2), Venezuela (2) and Paraguay (1). Source: clinicaltrials.gov.

The data presented in Figure 1 indicate the evolution of international multicenter clinical trial records sponsored by pharmaceutical companies in South American countries aiming to evaluate new cancer treatment drugs from 2010 to 2020. Figure 1A shows the total number of new clinical trials registered in South America during the 11-year study period. Interestingly, a 122% increase in the number of trials was identified. During the COVID-19 pandemic, the increased registration of new tests was maintained in relation to previous years, with the sum of new tests conducted in 2019 and 2020 totaling 24.95% (151) of the 605 clinical trials sponsored by foreign pharmaceutical companies in the region evaluated in the current study. Figure 1B marks a geographic investment border for clinical trials in the pharmaceutical production sector in South American countries, totaling 1251. Brazil and Argentina received the highest numbers of clinical trials, at 37.8% (473/1251) and 26.5% (332/1251), respectively, of the total number of new trials registered at clinicaltrials.gov for South America. Many of those studies were multicentric and, therefore, carried out in more than one South American country, which explains these values.

### 3.3. Number of Clinical Trials Distributed According to Clinical Phases

**Figure 2:**
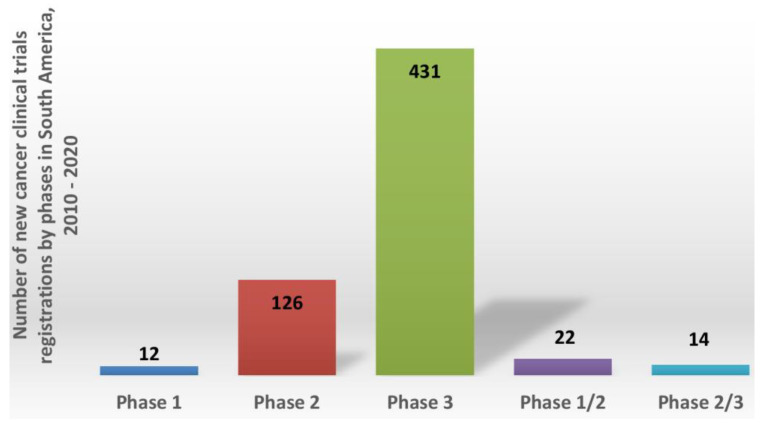
Number of new cancer clinical trial registrations per clinical phase in South America from 2010 to 2020 (source: clinicaltrials.gov).

The results depicted in Figure 2 indicate the number of clinical trials distributed in phases I, II and III for cancer drug development sponsored by foreign pharmaceutical companies in South America from 2010 to 2020. A prevalence of phase III clinical trials was noted, comprising 71.24% (431) of the total number of trials (605). Phase I trials were less prevalent, at only 2% of the total. These results indicate far more phase III studies than phase II studies (126, 30%), although the infrastructure requirements and professional skills for both are similar. The average duration of each clinical trial was 6 years, ranging from a minimum of 2 to a maximum of 10 years.

### 3.4. The Most Prevalent Pharmaceutical Companies That Sponsored Cancer Clinical Trials in South America

**Figure 3:**
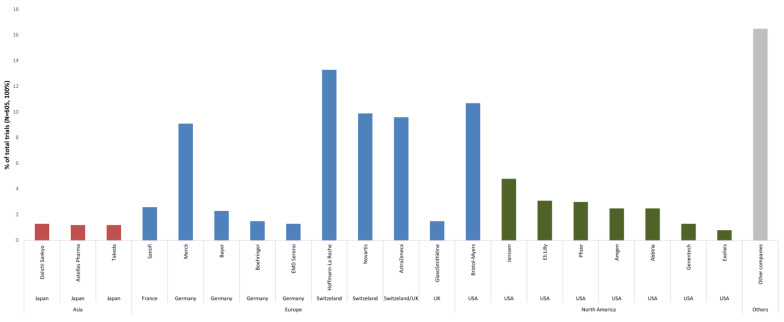
Ranking of the top 20 pharmaceutical companies that sponsored cancer clinical trials in South America, according to continent and country, from 2010 to 2020. Note: Sponsors conducting less than five clinical trials during the study period were omitted (other companies). Source: clinicaltrials.gov.

The profiles of pharmaceutical companies performing clinical studies in South America were investigated. The data shown in Figure 3 list the twenty pharmaceutical companies with the highest numbers of clinical trials in South America, totaling nine European, eight North American and three Asian countries. A complete list of names of the drugs used in the clinical trials analyzed can be found in Supplementary Table S1.

### 3.5. Clinical Research Interfaces

**Figure 4:**
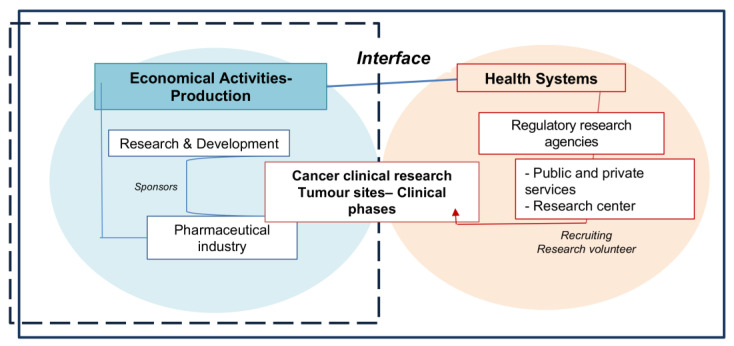
Clinical research interfaces (regulatory research agencies: Anmat: Argentina/Anvisa; Brazil/Anamed/ISP; Chile/Digemid; Peru/Invima; Colombia/Arcsa; Ecuador/DNVS; Paraguay/MSP; Uruguay/INH; RR/Venezuela).

The development process of new cancer drugs involves different factors, represented in Figure 4. This conceptual model portrays clinical research as an area/sector that acts interdependently with national and international clinical trial technological development, processing and management. Thus, a relationship was established between the domains portrayed in Figure 4.

## 4. Discussion

Clinical research process interfaces are based on relationships established between economically productive activities and health systems. The demographic and epidemiological transitions noted in recent decades have affected the distribution of chronic non-communicable diseases, such as cancer, in low- and middle-income countries, directly influencing new health technology research and development. This process has inspired activities concerning the testing of new cancer drugs in South America. Figure 1A,B demonstrate the interests of European and North American pharmaceutical companies in hiring clinical research centers in South America, especially for phase II and III clinical trials. This may be due to increasing South American population density and diversity, reaching 430 million inhabitants in 2020, as well as lower effective operating costs compared to economically developed countries [11], low local competition with respect to installed technological capacity and Universal Health Coverage (UHC), which exceeds 70% in nine of the thirteen member countries of this region [7,12].

Although it is a challenging research and development (R&D) area with high failure rates, oncology maintains a significant level of activity and remains one of the most significant health markets in the world; this was even the case during the COVID-19 pandemic. The increasing number of clinical trials registered from 2010 to 2020, in addition to demonstrating greater South American participation in conducting these studies, also reflects the expansion of cancer research and the search for personalized and more effective treatments. Globally, the pipeline of drugs at an advanced stage of development increased by 19% in 2018 alone and has increased by 63% since 2013 [13,14,15,16].

The current results point to the predominance of international pharmaceutical industries in the financing of clinical cancer research in South America. The contribution of the twenty industries with the highest numbers of clinical trials in the region amounts to 83.5% (505/605) of the total number of trials conducted during the study period. The present analysis also suggests regional productive base weaknesses in the search for cancer drug innovation, as South American pharmaceutical companies do not appear among the top twenty companies sponsoring new clinical trials. Efforts undertaken by South American governments to provide favorable conditions to attract international investments were noted, implemented through the opening and improvement of clinical research centers, physical infrastructure development, professional qualification and the creation of national regulatory drug authorities and regulations on research involving human subjects [17,18].

Interactions with public and/or private health services are required to assess pharmaceutical safety and efficacy. These services, with the aid of research centers, recruit study volunteers. Thus, a proportion of eligible patients presenting the most frequent cancers worldwide, such as lung and breast cancers, have the possibility of accessing new drugs under test conditions, although some tumors of public health interest in South American countries are still noted as representing a lower number of studies, such as cervical cancer (characterizing only 2.4% of the studies conducted from 2010 to 2020). Leukemia, for example, does not appear among the 10 most frequent cancer types in South America and occupies ninth place in the number of deaths but represents 6.8% of the studies registered from 2010 to 2020. Thus, there is a need to reflect on why this disease ranks higher than other cancers with higher incidence and mortality rates, such as stomach and pancreas cancers. This could be explained by pharmaceutical sector investment patterns over the years, which have leveraged financial resources for new drug discovery and testing for certain solid tumors such as breast, lung and prostate cancers, as well as onco-hematological diseases, including leukemia.

The number of compounds displaying potential for the treatment of colorectal, stomach and pancreas cancers, however, is lower, with a reduced number of clinical studies involving these diseases [19,20]. Nearly a third of drug indications approved between 2014 and 2018 were for hematological cancers such as leukemia, lymphoma and multiple myeloma, while lung cancer ranked first among solid tumors, with 12 indications, followed by melanoma and breast cancer [15].

The greater power of R&D investments depends directly on the results and profits generated through commercialized products. For example, Hoffman-La Roche had three products among the drugs with the highest sales revenue in 2019 (Avastin^®^, Herceptin^®^ and Mab Thera/Rituxan^®^) [21].

These data are accompanied by both opportunities and challenges, considering that technological changes are mounting in several areas, with social and economic impacts affecting the health goods and service productive sectors. This is especially true for the diagnosis and treatment of several types of cancer that make use of cutting-edge technologies, i.e., imaging tests, surgical procedures, radiotherapy and drugs [22,23]. A 66% increase in the number of cancer cases in low- and middle-income regions is predicted by 2030 based on population aging and growth [5]. This will entail enhancing the local research workforce to investigate important issues and test cancer control interventions in South America. This will require the implementation of a collaborative system with the union of South American countries, as research advances confront problems involving access to health and high-quality treatment, which is known to be fragile in South America. Therefore, it is essential to align health systems with disease inputs and burdens. However, the GDP health investment in South American countries is still lower compared to that in countries in more developed regions. Argentina and Brazil, for example, invest 9.6% and 9.5% of their Gross Domestic Product (GDP) in health, respectively, while the United States invests 18% [13].

According to data from the World Bank, Brazil, Argentina and Chile invested approximately 1.2%, 0.6% and 0.3% of their Gross Domestic Product (GDP) in research and development in the year 2013, respectively. This trend is believed to be similar for other years under analysis [24]. It is evident that a greater allocation of resources to R&D tends to contribute to the strengthening of this sector and a consequent increase in the capacity for expanding clinical research in this region. In addition to other factors, these differences in the availability of GDP resources may partially explain the distribution of clinical trials represented in Figure 1B. This scenario indicates the need for greater investment to achieve practical qualification effects and a consequent increase in the region’s health productivity. Each country within the South American bloc should have sufficient resources for the development of basic and clinical research on the regional level, as well as the planning and execution of collaborative actions for the incorporation of new technologies in cancer treatment. In cancer research, these resources would be distributed to ensure the progression of local research, the improvement of operational infrastructure (equipment and facilities), the training and education of qualified professionals, the construction of more clinical research centers and other strategic actions aimed towards developing studies for cancer control in this region [25,26,27,28].

Another important discussion concerns equitable access to new drugs, which has been debated even in high-income countries such as the United States and the United Kingdom. Several proposals have been created to guarantee both company profits and accessibility to both public and private health insurers. One of the fundamental principles in this regard is that governments and industries must work together to align their interests, such as funding and regulations that focus on patient needs [16,29].

The cost of cancer treatment drugs has significantly increased over the years, rising from USD 12,000 before the year 2000 to over USD 120,000 by 2015, which exceeds the per capita GDP of South American countries [30,31]. Some suggestions were proposed in a previous study to address this scenario of limited resources, such as the incorporation of cost-effectiveness analyses of new health technologies to enable negotiation, financing and the acquisition of new products at differentiated prices. This includes the use of adapted therapeutic regimens, generic drugs and high-quality biosimilar drugs, as well as the performance of clinical trials [32].

The limitations of this study comprise the low number of available records on the cancer clinical studies of certain South American countries, such as Ecuador (5), Uruguay (2), Venezuela (2) and Paraguay (1), in the clinicaltrials.gov database, in addition to the low number of publications regarding the data from these countries; a larger publication base would add more knowledge to complete our analysis.

The offer of new cancer treatments involves research investments, innovation and health service strengthening. The involvement of South American countries in new cancer treatment drug testing processes is one of the important strategies applied to link different sectors such as health, economy, science and technology.

## 5. Conclusions

This study reinforces the notion that oncology R&D activities in Brazil are under the management of foreign biotechnology companies and pharmaceutical industries, which have greater investment power concerning cancer clinical trials. Thus, the inclusion of South American clinical research activity participants guarantees contact between research centers offering innovative treatments and allows volunteers to access certain novel drugs.

Although South American health services are important recruiters of volunteers for studies, local operational and financial health service weaknesses may render high-quality treatments unfeasible in the future. It is possible that many of the newly developed drugs, after their approval, will become inaccessible to local populations due to their high marketing costs. Therefore, the data reported herein indicate the need for strategic basic and clinical research planning that considers South American epidemic cancer profiles, as an imbalance between the demand for innovative treatments and their public health system availability for cancer-affected populations in South America is noteworthy.

## Figures and Tables

**Table 1:** Incidence and mortality rates for the top 10 most common cancers and all other cancers combined for 10 South American countries in 2020.

Cancer Site	Incidence	(%)	Cancer Site	Deaths	(%)
Breast	156,021	14.3	Lung	67,145	12.9
Prostate	155,875	14.3	Colorectum	51,876	10.0
Colorectum	103,725	9.5	Breast	41,543	8.0
Lung	76,416	7.0	Stomach	39,102	7.5
Stomach	49,460	4.5	Prostate	37,266	7.2
Thyroid	47,903	4.4	Pancreas	27,055	5.2
Cervix uteri	41,517	3.8	Liver	23,071	4.4
Non-Hodgkin lymphoma	28,017	2.6	Cervix uteri	22,101	4.3
Pancreas	27,962	2.6	Leukemia	18,636	3.6
Kidney	27,484	2.5	Esophagus	14,439	2.8
Others	378,198	34.6	Others	177,713	34.2

Note: Included countries: Argentina, Bolivia, Brazil, Chile, Colombia, Ecuador, Paraguay, Peru, Uruguay and Venezuela. Source: GLOBOCAN, 2020 [4].

**Table 2:** Clinical trials registered in South America from 2010 to 2020.

Cancer Site by Clinical Trial	N = 620	%	Continuation	N.	%
Lung	119	19.2	Liver	16	2.6
Breast	100	16.1	Cervix uteri	15	2.4
Leukemia	42	6.8	Ovary	14	2.3
Prostate	39	6.3	Central Nervous system (CNS); brain	8	1.3
Melanoma	32	5.2	Pancreas	7	1.1
Non-Hodgkin lymphoma	29	4.7	Corpus uteri	6	1.0
Stomach	25	4	Soft tissue sarcoma	5	0.8
Head and neck; thyroid; pral cavity	23	3.7	Biliary tract	5	0.8
Kidney	22	3.5	Hodgkin lymphoma	4	0.6
Bladder	21	3.4	Mesothelioma	2	0.3
Colorectal	20	3.2	Non-melanoma skin cancer	1	0.2
Multiple myeloma	20	3.2	Tumor site not reported	20	3.2
Esophagus	19	3.1	Different types of cancer	6	1.0

Note: Some studies include more than one tumoral site. Source: clinicaltrials.gov.

## Data Availability

Publicly available datasets were analyzed in this study as referenced in the methodology section.

## Supplementary Materials

The following supporting information can be downloaded at: https://www.mdpi.com/article/10.3390/healthcare11121753/s1, Supplementary Table S1. List of drugs that have been used in the trials analyzed (N = 305).
