# Supplementary material for: Clinical Cancer Research in South America and Potential Health Economic Impacts
Source: Healthcare (Basel). 2023 Jun 15;11(12):1753. doi: 10.3390/healthcare11121753 (PMC10298265; doi:10.3390/healthcare11121753)
Supplement: Supplementary file 1 [file healthcare-11-01753-s001.zip › healthcare-2345966-supplementary.pdf]

Supplementary Table S1. List of drugs that have been used in the trials analyzed (N=305).

Drug name	Drug type
5-Fluouracil	Chemotherapy
Abemaciclib	Chemotherapy
Abiraterone acetate	Chemotherapy
Acalabrutinib	Chemotherapy
Ado-trastuzumab emtansine	Imunotherapy
Afatinib	Targeted therapy
Afatinib (BIBW-2992)	Targeted therapy
Aflibercept	Chemotherapy
AIP-303	Targeted therapy
Aldoxorubicin	Chemotherapy
Alpelisib	Targeted therapy
Amcenestrant (SAR439859)	Chemotherapy
AMG 337	Targeted therapy
Amivantamab	Imunotherapy
Amrubicin	Chemotherapy
Anastrozole	Hormonal Therapy
Andecaliximab	Imunotherapy
Andes-1537	Targeted therapy
Apalutamide	Chemotherapy
Apitolisib (GDC-0980)	Targeted therapy
Asciminib (ABL 001)	Targeted therapy
Atezolizumab	Imunotherapy
Avelumab	Imunotherapy
Axalimogene filolisbac (ADXS11-001)	Imunotherapy
Axitinib	Targeted therapy
Azacitidine	Chemotherapy
Balixafortide	Chemotherapy
Balstilimab (AGEN2034)	Imunotherapy
Belantamab mafodotin	Imunotherapy
Belzutifan	Chemotherapy
Bempegaldesleukin	Imunotherapy
Bendamustine	Chemotherapy
bevacizumab	Imunotherapy
Bevacizumab BEVZ92	Imunotherapy
Bevacizumab biosimilar (BI 695502)	Imunotherapy
Bevacizumab biosimilar (MB02)	Imunotherapy
Bexarotene	Chemotherapy
Bicalutamide	Hormonal Therapy
Binimetinib	Targeted therapy

Bintrafusp	Targeted therapy
Bintrafusp alfa	Targeted therapy
Bleomycin	Chemotherapy
Blinatumomab	Imunotherapy
BMS-690514	Targeted therapy
BMS-833923	Targeted therapy
BMS-986218	Imunotherapy
BMS-986315	Imunotherapy
Bortezomib	Targeted therapy
Bosutinib	Targeted therapy
Brentuximab vedotin	Imunotherapy
Brigatinib	Targeted therapy
Buparlisib	Targeted therapy
Buparlisib (BKM120)	Targeted therapy
Cabazitazel	Chemotherapy
Cabozantinib	Targeted therapy
Canakinumab	Imunotherapy
Capecitabine	Chemotherapy
Capivasertib	Targeted therapy
Capivasertib (AZD5363)	Targeted therapy
Capmatinib	Targeted therapy
Carboplatin	Chemotherapy
Carfilzomib	Targeted therapy
Cemiplimab	Imunotherapy
Ceritinib	Targeted therapy
Cetrelimab	Imunotherapy
Cetuximab	Imunotherapy
Chlorambucil	Chemotherapy
CHOP	Chemotherapy
CIGB300	Targeted therapy
Cisplatin	Chemotherapy
Citoplurikin (IRX2)	Imunotherapy
Cixutumumab	Imunotherapy
Cobimetinib	Targeted therapy
Cobolimab	Imunotherapy
Copanlisib	Targeted therapy
Crizotinib	Targeted therapy
Cusatuzumab	Imunotherapy
Cyclophosphamide	Chemotherapy
Cytarabine	Chemotherapy
Dabrafenib	Targeted therapy
Dacarbazine	Chemotherapy
Dactolisib (BEZ235)	Targeted therapy

Daratumumab	Imunotherapy
Darolutamide	Chemotherapy
Darolutamide (BAY1841788)	Hormonal Therapy
Dasatinib	Targeted therapy
Datopotamab	Imunotherapy
Daunorubicin	Chemotherapy
Daunorubicin liposomal	Chemotherapy
Debio 1347	Targeted therapy
Denosumab	Imunotherapy
Depatuxizumab mafodotin (ABT-414)	Imunotherapy
Derazantinib	Targeted therapy
Docetaxel	Chemotherapy
Dostarlimab	Imunotherapy
Dovitinib	Targeted therapy
Dovitinib	Targeted therapy
Doxorubicin	Chemotherapy
Durvalumab	Imunotherapy
E7820	Targeted therapy
Elacestrant	Chemotherapy
Enasidenib (AG-221)	Targeted therapy
Encorafenib	Targeted therapy
Encorafenib	Targeted therapy
Ensartinib	Targeted therapy
Entrectinib	Targeted therapy
Enzalutamide	Hormonal Therapy
Epacadostat	Imunotherapy
Epirubicin	Chemotherapy
Erbitux	Chemotherapy
Erdafitinib	Targeted therapy
Eribulin	Chemotherapy
Erlotinib	Targeted therapy
Etoposide	Chemotherapy
Everolimus	Targeted therapy
Exemestane	Hormonal Therapy
Feladilimab	Imunotherapy
Fludarabine	Chemotherapy
Fulvestrant	Hormonal Therapy
Ganetespib	Targeted therapy
Ganitumab (AMG-479)	Imunotherapy
Gebasaxturev	Virotherapy
Gefitinib	Targeted therapy
Gemcitabine	Chemotherapy
Gencitabine (CO-1.01)	Chemotherapy

Gevokizumab	Imunotherapy
Gilteritinib	Targeted therapy
Giredestrant	Hormonal Therapy
Goserelin	Hormonal Therapy
Gusacitinib (ASN002)	Imunotherapy
Ibrutinib	Chemotherapy
Idarubicin	Chemotherapy
Ieramilimab	Imunotherapy
Ifosfamide	Chemotherapy
Imatinib	Targeted therapy
Inotuzumab ozogamicin	Imunotherapy
Ipatasertib	Chemotherapy
Ipilimumab	Imunotherapy
Irinotecan	Chemotherapy
Irinotecan Liposomal	Chemotherapy
Isatuximab	Imunotherapy
Ivosidenib	Targeted therapy
Ixazomib	Targeted therapy
Keyhole limpet hemocyanin (KLH)	Imunotherapy
Lapatinib	Targeted therapy
Larotrectinib	Targeted therapy
L-asparaginase	Chemotherapy
Lazertinib	Targeted therapy
LCL161	Targeted therapy
Lenalidomide	Imunotherapy
Lenvatinib	Targeted therapy
Letrozole	Hormonal Therapy
Leucovorin	Chemotherapy
Leuprolide	Hormonal Therapy
Linifanib (ABT-869)	Targeted therapy
Linrodostat (BMS-986205)	Imunotherapy
Linsitinib	Targeted therapy
Liposomal daunorubicin	Chemotherapy
Lorlatinib	Targeted therapy
Lurbinectedin	Chemotherapy
Melphalan	Chemotherapy
Merestinib	Targeted therapy
Methotrexate	Chemotherapy
Midostaurin	Chemotherapy
Mitomycin C	Chemotherapy
Mitoxantrone	Chemotherapy
Mogamulizumab	Imunotherapy
Monalizumab	Imunotherapy

Monoclonal antibody Hu3S193	Targeted therapy
Montelukast	Imunotherapy
Nab-paclitaxel	Chemotherapy
Nanoliposomal irinotecan	Chemotherapy
Napabucasin (BBI608)	Targeted therapy
Naquotinib mesilate	Targeted therapy
Navitoclax (ABT-263)	Targeted therapy
Necitumumab	Imunotherapy
Neratinib	Targeted therapy
Nilotinib	Targeted therapy
Nimotuzumab	Imunotherapy
Nintedanib	Targeted therapy
Niraparib	Targeted therapy
Nivolumab	Imunotherapy
Nivolumab/Relatlimab (BMS-986213)	Imunotherapy
Obinutuzumab	Imunotherapy
Ofatumumab	Imunotherapy
Olaparib	Targeted therapy
Olaratumab	Imunotherapy
Ombrabulin	Targeted therapy
Omidubicel	Cell therapy
Onartuzumab	Imunotherapy
Onartuzumab	Imunotherapy
Oraxol	Chemotherapy
Oregovomab	Imunotherapy
Orteronel	Hormonal Therapy
Osimertinib	Targeted therapy
Osimertinib (AZD9291)	Targeted therapy
Oxaliplatin	Chemotherapy
Paclitaxel	Chemotherapy
Palbociclib	Targeted therapy
Palifosfamide-tris	Chemotherapy
Panitumumab	Imunotherapy
Panobinostat	Targeted therapy
Pasireotide	Hormonal Therapy
Patritumab	Imunotherapy
Pazopanib	Targeted therapy
PEG-asparaginase	Targeted therapy
Pembrolizumab	Imunotherapy
Pemetrexed	Chemotherapy
Pertuzumab	Imunotherapy
Pevonedistat	Targeted therapy
Pictilisib	Targeted therapy

Pictilisib (GDC-0941)	Targeted therapy
Polatuzumab Vedotin	Imunotherapy
Pomalidomide	Imunotherapy
Ponatinib	Targeted therapy
Pracinostat	HDAC Inhibitors
Pralatrexate	Chemotherapy
Prednisone	Supportive medicine
Quavonlimab	Imunotherapy
Quizartinib	Targeted therapy
Racotumomab	Imunotherapy
Radium-223 dichloride	Radioactive isotop
Ramucirumab	Imunotherapy
Regorafenib	Targeted therapy
Relatlimab	Imunotherapy
Relugolix	Hormonal therapy
Ribociclib	Targeted therapy
Ribociclib	Targeted therapy
Rilotumumab	Imunotherapy
Rindopepimut	Imunotherapy
Ripretinib (DCC-2618)	Targeted therapy
Rituximab	Imunotherapy
Rituximab biosimilar (ABP 798)	Imunotherapy
Rituximab biosimilar (GP2013)	Imunotherapy
Rituximab biosimilar (PF-05280586)	Imunotherapy
Rituximab biosimilar (RTXM83)	Imunotherapy
Rituximab biosimilar (SAIT101)	Imunotherapy
Romidepsin	HDAC Inhibitors
Rovalpituzumab tesirine	Imunotherapy
Rucaparib	Targeted therapy
Ruxolitinib	Targeted therapy
Sapitinib (AZD-8931)	Targeted therapy
Savolitinib	Targeted therapy
Selpercatinib	Targeted therapy
Selumetinib	Targeted therapy
Sonidegib	Targeted therapy
Sorafenib	Targeted therapy
Sotorasib (AMG 510)	Targeted therapy
Sotorasib (AMG 510)	Targeted therapy
Spartalizumab	Imunotherapy
Sunitinib	Targeted therapy
Tabalumab	Imunotherapy
Talazoparib	Targeted therapy
Talimogene Laherparepvec	Gene Therapy

Tamoxifen	Hormonal Therapy
TAR-200	Chemotherapy
Taselisib	Targeted therapy
Tasquinimod	Immunomodulatory agent
Tegafur/Gimeracil/Oteracil	Chemotherapy
Temozolomide	Chemotherapy
Temsirolimus	Targeted therapy
Thalidomide	Imunotherapy
Tipiracil hydrochloride	Chemotherapy
Tiragolumab	Imunotherapy
Tisagenlecleucel	Targeted therapy
Tislelizumab	Imunotherapy
Tivantinib	Targeted therapy
Tivozanib	Targeted therapy
Tizanidine	Targeted therapy
Topotecan	Chemotherapy
Trabectedin	Chemotherapy
Trametinib	Targeted therapy
Trastuzumab	Imunotherapy
Trastuzumab biosimilar (ABP 980)	Imunotherapy
Trastuzumab biosimilar (EG12014)	Imunotherapy
Trastuzumab biosimilar (MYL- 1401O)	Imunotherapy
Trastuzumab biosimilar (PF-05280014)	Imunotherapy
Trastuzumab biosimilar (TX05)	Imunotherapy
Trastuzumab deruxtecan	Imunotherapy
Trastuzumab emtansine	Imunotherapy
Trebananib (AMG-386)	Targeted therapy
Tremelimumab	Imunotherapy
Trifluridine	Chemotherapy
Trifluridine/Tipiracil hydrochloride	Chemotherapy
Tucatinib	Targeted therapy
Tusamitamab ravtansine	Imunotherapy
Tusamitamab ravtansine	Imunotherapy
Ulevostinag	Imunotherapy
Ulocuplumab (BMS-936564)	Imunotherapy
Vandetanib	Targeted therapy
Velaparib	Targeted therapy
Vemurafenib	Targeted therapy
Venetoclax	Targeted therapy
VGX-3100	Vaccine / Imunotherapy
Vinblastine	Chemotherapy
Vinflunine	Chemotherapy
Vinorelbine	Chemotherapy

Vinscritine	Chemotherapy
Vismodegib	Targeted therapy
Volasertib	Targeted therapy
Xevinapant (Debio 1143)	Chemotherapy
XmAb24306	Imunotherapy
Zalifrelimab (AGEN1884)	Imunotherapy
Zanidatamab	Imunotherapy
Zolbetuximab	Imunotherapy

Source: clinicaltrials.gov
